# Isochromanoindolenines suppress triple-negative breast cancer cell proliferation partially via inhibiting Akt activation

**DOI:** 10.7150/ijbs.48170

**Published:** 2021-03-02

**Authors:** Xiaoyan Jiang, Xu Zhi, Peixia Zhang, Zhongmei Zhou, Jinxiang Ye, Yu Gao, Xinye Wang, Chuanyu Yang, Haijun Chen, Rong Liu, Ceshi Chen

**Affiliations:** 1 Medical Faculty of Kunming University of Science and Technology, Kunming 650500, China.; 2 Key Laboratory of Animal Models and Human Disease Mechanisms of the Chinese Academy of Sciences and Yunnan Province, Kunming Institute of Zoology, Chinese Academy of Sciences, Kunming, 650223, China.; 3 Center for Reproductive Medicine, Department of Obstetrics and Gynecology, Peking University Third Hospital, Beijing, 100191, China.; 4 College of Chemistry, Fuzhou University, Fuzhou, Fujian 350116, China.; 5 Translational Cancer Research Center, Peking University First Hospital, Beijing, 100034, China.; 6 KIZ-CUHK Joint Laboratory of Bioresources and Molecular Research in Common Diseases, Kunming Institute of Zoology, Chinese Academy of Sciences, Kunming, 650223, China.; 7 Institute of Translation Medicine, Shenzhen Second People's Hospital, The First Affiliated Hospital of Shenzhen University, Shenzhen 518035, China.

**Keywords:** Isochromanoindolenine, TNBC, cell cycle arrest, AKT

## Abstract

As the most malignant subtype of breast cancers, triple-negative breast cancer (TNBC) lacks effective targeted therapeutics clinically to date. In this study, one lead compound FZU-0025-065 with isochromanoindolenine scaffold was identified by a cell-based screening. Among nine breast cancer cell lines tested, TNBC are the most sensitive cell lines to FZU-0025-065. FZU-0025-065 inhibits TNBC cell growth in a time- and dosage-dependent manner. FZU-0025-065 suppresses the expression of cell cycle dependent kinase 4 (CDK4), Cyclin D1 and Cyclin B1; meanwhile, elevates the expression of cell cycle dependent kinase inhibitor p21 and p27. Importantly, we found that FZU-0025-065 suppresses AKT activation in a time- and dosage-dependent manner. Over-expression of constitutive active AKT partially rescues FZU-0025-065 induced cell growth inhibition in MDA-MB-468 cells, indicating FZU-0025-065 suppresses TNBC cell growth partially via inhibiting AKT activation. Finally, FZU-0025-065 suppresses TNBC cell growth in a xenograft mouse model. Taken together, our findings suggested that isochromanoindolenine derivative FZU-0025-065 inhibits TNBC via suppressing the AKT signaling and that FZU-0025-065 may be useful for TNBC treatment.

## Introduction

Triple-negative breast cancer (TNBC) is defined by the absence of expression of the estrogen receptor (ERα), progesterone receptor (PR), and human epidermal growth factor receptor 2 (HER2), and accounts for approximately 10-15% of all breast cancers. Compared to other subtypes, TNBC has higher rates of metastases and relapse. Recently, two poly ADP-ribose polymerase (PARP) inhibitors, Olaparib and Talazoparib, have been approved for TNBC patients with germline breast cancer susceptibility gene (BRCA) mutations 1,2. However, these drugs could not extend patient overall survival. Additionally, atezolizumab (an anti-PD-L1 mAb) and Nab-paclitaxel have been approved for PD-L1 positive advanced TNBC patients although the overall response rate is low 3. Thus, it's still important and urgent to find effective drugs to treat such disease.

AKT is the essential mediator of phosphoinositide 3 kinase (PI3K) pathway, which is frequently activated in breast cancer 4. Activated AKT signaling has been well-documented to promote tumor initiation, cell proliferation, survival, angiogenesis, and metastasis 5-10. AKT has been reported to promote cell proliferation via downregulating p21^Cip1/WAF1^ and/or p27^Kip1^ expression 11-14. Meanwhile, cell cycle regulators cyclin D1 and cyclin B1 also play important roles in mediating AKT's functions in promoting cancer cell proliferation 15,16. Efforts have been put in studying AKT inhibitors for the treatment of breast cancer 17-19; however, no AKT inhibitors have been approved for clinical application so far although the PI3Kα inhibitor Alpelisib in combination with Fulvestrant has been approved for ERα-positive and HER2-negative breast cancer patients with PIK3CA gene mutation 20.

Isochromanoindolenines are core scaffolds of certain natural alkaloids, such as bipleiophyline, a complex monoterpene indole alkaloid isolated from *Alstonia angustifolia* 21, or voacalgine A extracted from *Voacanga grandifolia* 22. A diverse chemical library of isochromanoindolenines was constructed through the last-stage functionalization of tetrahydro-β-carbolines (THβC) via biomimetic oxidative coupling cyclization 23. The suitable calculated physicochemical properties of this skeletally diverse chemical library suggest that these isochromanoindolenine derivatives are worthwhile for biological evaluation and pharmacological characterizations.

In this study, we first screened anti-cancer activities of the 23 isochromanoindolenines in breast cancer cells and identified FZU-0025-065 to be the most potent one. Interestingly, FZU-0025-065 showed the strongest cytotoxic effects in TNBCs. We found FZU-0025-065 significantly suppressed TNBC cell growth in MDA-MB-468 and HCC1806 TNBC cells. FZU-0025-065 reduced the protein expression levels of CDK4, cyclin D1, cyclin B1, and increased the expression of p21 and p27. We also found that FZU-0025-065 significantly suppressed AKT activation in TNBC cells. Furthermore, ectopic overexpression of constitutively active AKT partially rescued FZU-0025-065 induced cyclin D1 and cyclin B1 reduction and cell growth inhibition. These findings suggest that FZU-0025-065 is a cell proliferation inhibitor and may be an effective anticancer agent for the treatment of TNBC.

## Results

### FZU-0025-065 is the most potent anti-cancer compound among the 23 tested Isochromanoindolenine derivatives

**Figure 1:**
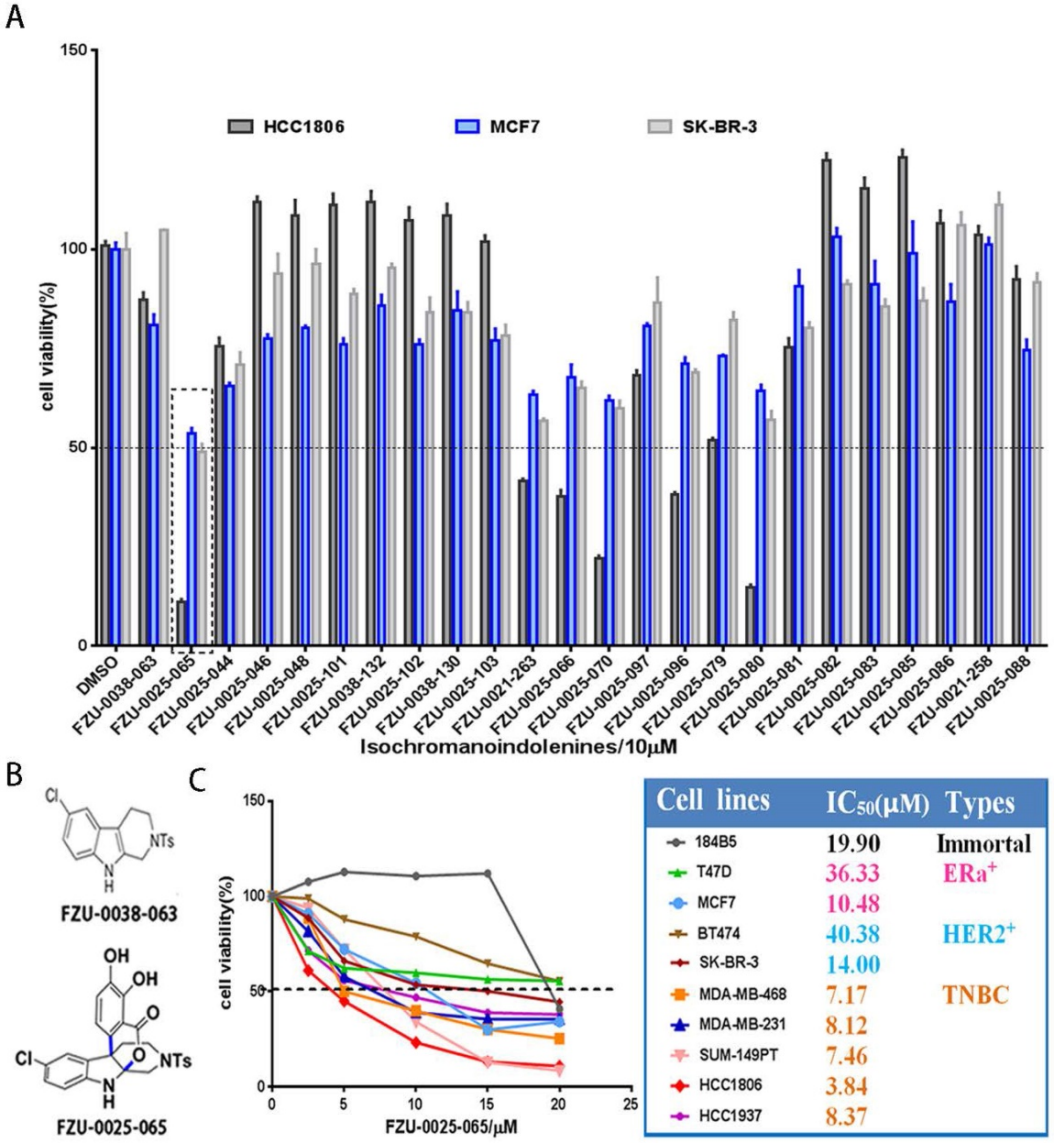
** FZU-0025-065 was identified as a more potent anti-cancer compound derived from isochromanoindolenine in breast cancer cells.** (A) Effects of new isochromanoindolenine derivatives (10 µM) on cell survival of breast cancer cell lines HCC1806, MCF7 and SK-BR-3. (B) The structure of FZU-0025-065 and its precursor FZU-0038-063, a synthesized THβC. (C) The cytotoxicity of FZU-0025-065 in 9 different breast cancer lines. The cells were treated with FZU-0025-065 at indicated dosages for 48 hours and cell viabilities were measured using the SRB assay.

We first investigated the cytotoxic potency of these newly synthesized isochromanoindolenine derivatives (Supplementary Table 1) in TNBC cell line HCC1806, ERα+ cell line MCF7, and HER2+ cell line SR-BR-3 using SRB assays. As the results shown in Figure 1A, among the 23 compounds, FZU-0025-065, which is one of optimal isochromanoindolenines scaffolds synthesized by oxidatively coupling catalyzation from FZU-0038-063, the THβC scaffold (Fig. 1B), showed the most potent cytotoxicity against these cancer cell lines, especially the TNBC cell line HCC1806. Then, we examined the half maximal inhibitory concentrations (IC_50_) of FZU-0025-065 in five TNBC cell lines (HCC1806, HCC1937, MDA-MB-468, MDA-MB-231 and SUM-149PT), two ERα positive breast cancer cell lines (MCF-7 and T47D), two HER2 positive breast cancer cell lines (BT474 and SK-BR-3), and the human immortalized breast epithelial cell line 184B5. As the data shown in Figure 1C, TNBC is the most sensitive subtype for FZU-0025-065 among all tested breast cell subtypes. FZU-0025-065 suppressed TNBC cell survival with the IC_50_s no more than 8.5 μM, while it suppressed other subtype breast cell survival with IC_50_s 10.48-40.38 μM.

### FZU-0025-065 suppresses cell proliferation in TNBC cells

**Figure 2:**
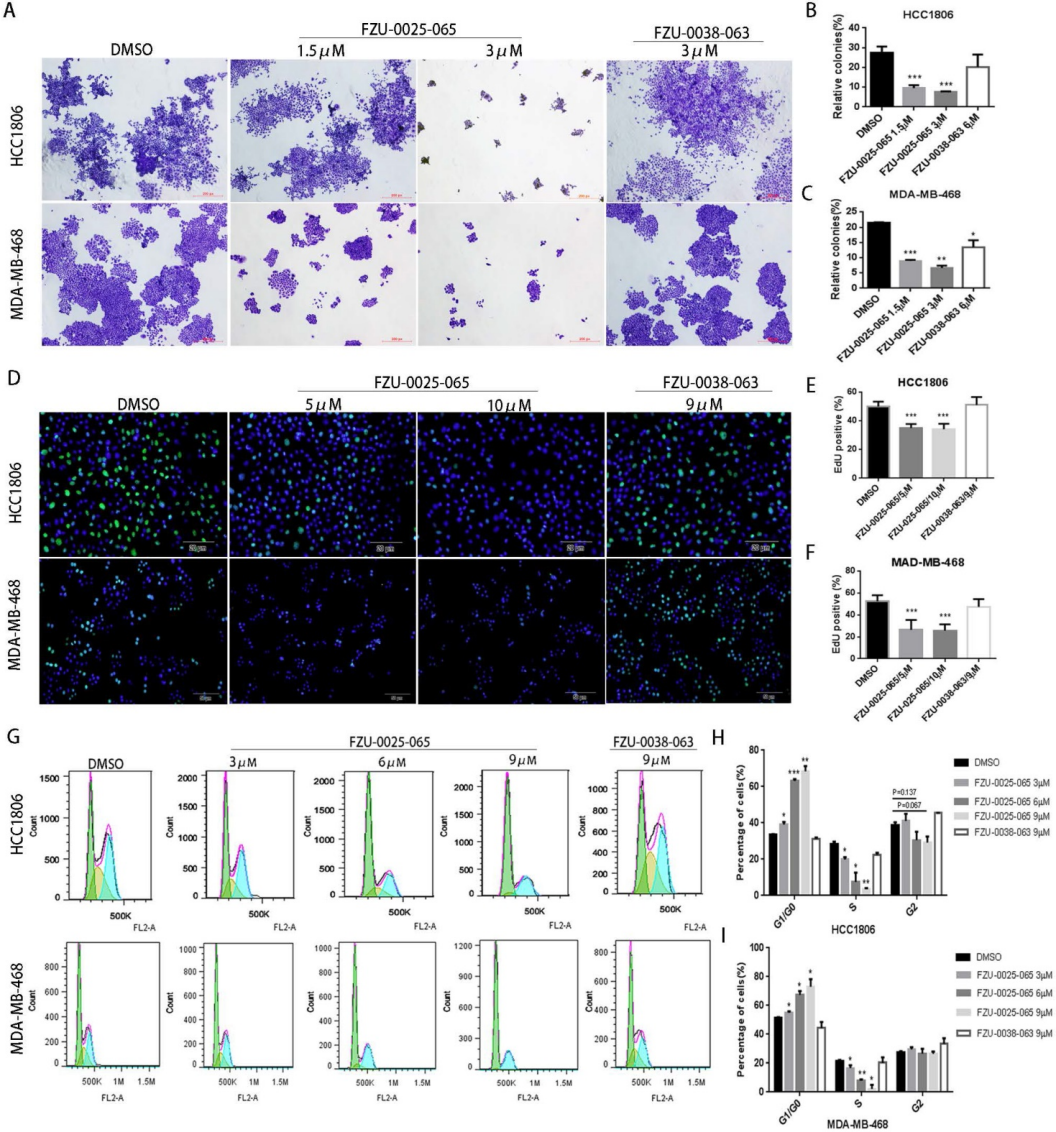
** FZU-0025-065 suppresses cell growth in TNBC cells.** (A-C) FZU-0025-065 suppresses colony formation in HCC1806 and MDA-MB-468 TNBC cells. HCC1806 and MDA-MB-468 cells were plated in 6-well plate at a concentration of 5,000 cells/well, followed by treating with either FZU-0025-065, FZU-0038-063 or DMSO control for 3 weeks. Cells were then fixed for crystal violet colony formation assay (A). The quantitative data of HCC1806 (B) and MDA-MB-468 (C) were shown on the right. (D-F) FZU-0025-065 inhibits DNA synthesis in HCC1806 and MDA-MB-468 cells. DNA synthesis of FZU-0025-065, FZU-0038-063 or DMSO treated cells were examined using the cell-Light^TM^ EdU Apollo488 *In vitro* Kit (D). The quantitative results of HCC1806 (E) and MDA-MB-468 (F) were shown on the right. (G-I) FZU-0025-065 suppresses HCC1806 and MDA-MB-468 cell cycle progression in a dosage-dependent manner. HCC1806 and MDA-MB-468 cells were treated with FZU-0025-065, FZU-0038-063 or DMSO at indicated dosage for 24 hours. The cells were then collected for cell cycle analysis. The quantitative results of HCC1806 (H) and MDA-MB-468 (I) were shown on the right, *p<0.5, **p<0.01, ***p<0.001, t-test.

It is known that both cell growth inhibition and apoptosis reduce cell viability. We investigated the effects of FZU-0025-065 on cell growth and apoptosis in two TNBC cell lines HCC1806 and MDA-MB-468. Cell growth was assessed using the colony formation assay. FZU-0025-065 significantly inhibited colony formation in a dosage-dependent manner (Fig. 2A-C) compared to FZU-0038-063 and DMSO controls. Following that, we confirmed that FZU-0025-065 inhibits cell proliferation using EdU incorporation assays and cell cycle analyses. As the data shown in Figure 2D-F, FZU-0025-065 suppressed DNA synthesis in a dosage-dependent manner in both HCC1806 and MDA-MB-468 cell lines. FZU-0025-065 also suppressed breast cancer cell cycle progression by arresting cells in G1/G0 phase (Fig. 2G-I). Consistently, the S-phase cell populations were significantly decreased by FZU-0025-065. We then performed Annexin V staining and flow cytometry analyses in HCC1806 and MDA-MB-468 cells after FZU-0025-065 treatment. However, FZU-0025-065 only moderately induced apoptosis in HCC1806, while it did not induce obvious apoptosis in MDA-MB-468 (Supplementary Figure 1).

### FZU-0025-065 regulates cell cycle-related proteins' expression in TNBC cells

**Figure 3:**
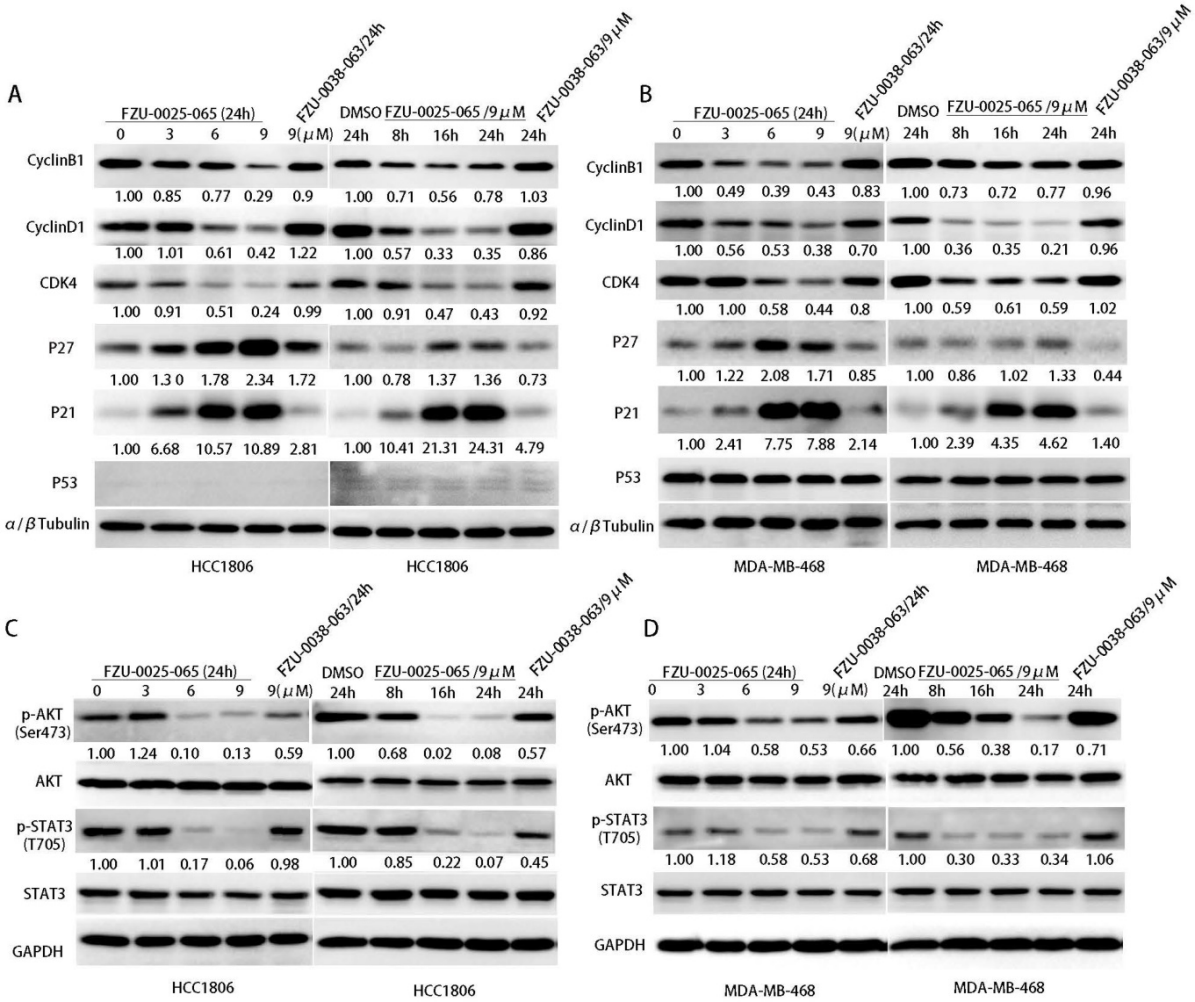
** FZU-0025-065 regulates cell cycle associated proteins' expression and suppresses AKT activation.** (A-B) FZU-0025-065 suppresses the expression CDK4, cyclin B1, cyclin D1, and promotes the expression of cell cycle-dependent kinase inhibitor p21, p27. HCC1806 and MDA-MB-468 cells were treated with FZU-0025-065, FZU-0038-063 or DMSO control for indicated time and at indicated dosages. The α,β-tubulin was detected as loading control. (C-D) FZU-0025-065 inhibits AKT activation. HCC1806 and MDA-MB-468 cells were treated with FZU-0025-065, FZU-0038-063 or DMSO control for indicated time and at indicated dosages. GAPDH was detected as loading control.

Cell cycle progression is facilitated by cyclin-dependent kinases that are activated by cyclins and inactivated by cyclin-dependent kinase inhibitors (CDKIs). Since FZU-0025-065 inhibits cell proliferation and cell cycle progression in HCC1806 and MDA-MB-468 cells, we examined the expression of cell cycle related proteins by western blot analysis. FZU-0025-065 decreased the expression of CDK4, cyclin D1 and cyclin B1 in the HCC1806 and MDA-MB-468 cell lines in time- and dose- dependent manners (Fig. 3A-B). Meanwhile, it significantly increased the protein expression of CDKIs, p21 and p27 (Fig. 3A-B).

Since FZU-0025-065 regulates the expression of several cell cycle-related proteins, we further examined several oncogenic signaling pathways, including AKT, MAPK, STAT3, and p53, etc. We found that the phosphorylated AKT (Ser473) is consistently suppressed by FZU-0025-065 in time- and dose-dependent manner in both HCC1806 and MDA-MB-468 cells (Fig. 3C-D).

### FZU-0025-065 suppresses TNBC cell proliferation partially by inhibiting AKT

**Figure 4:**
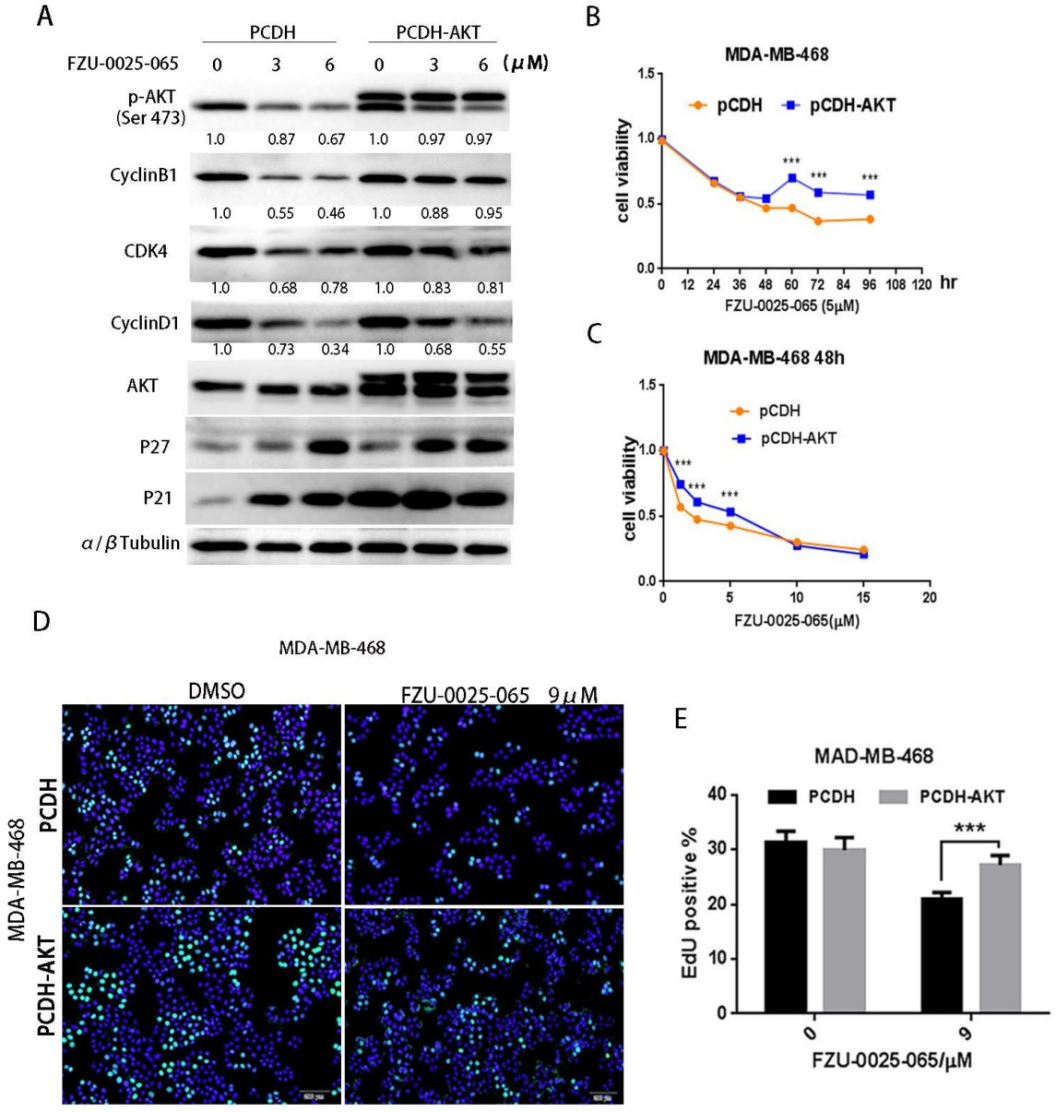
** FZU-0025-065 suppresses TNBC cell cycle progression partially through inhibiting AKT signaling.** (A) Ectopic overexpression of AKT partially rescued FZU-0025-056 caused reduction of cyclin D1, cyllin B1 and CDK4. (B-C) Ectopic overexpression of AKT partially rescued FZU-0025-056 caused cell survival inhibition. MDA-MB-468 cells were plated in 48-well plate at a concentration of 2×10^4^ cells/well. 24 hours after plating, cells were treated with FZU-0025-056 at 5 µM for indicated time (B) and indicated dosages for 48 hours (C). Cells were then collected for SRB assay. (D-E) Ectopic overexpression of AKT partially rescued FZU-0025-056 caused cell growth inhibition. MDA-MB-468 cells were plated and treated with FZU-0025-056 at indicated dosages for 24 hours. DNA synthesis of FZU-0025-056 treated cells was examined using the cell-Light^TM^ EdU Apollo488 *In vitro* Kit (C). The quantitative results are shown on the right (D). All experiments have been performed three times independently, representative data are shown as mean±SD, *p<0.5.

To test whether AKT mediates the cell growth inhibition function of FZU-0025-065 in TNBC cells, we overexpressed a constitutive form of AKT in MDA-MB-468 (Fig. 4A) and HCC1806 (Supplementary Figure 2) cells. Ectopic overexpression of AKT obviously restored the expression levels of cyclin D1, cyclin B1, and CDK4 in the presence of FZU-0025-065 (Fig. 4A and Supplementary Figure 2). Considering FZU-0025-065 inhibits TNBC cell in G1/G0 phase, while does not affect cell distribution in G2/M phase significantly (Fig. 2G-I), cyclin D1 might play more important roles in mediating FZU-0025-065's cell growth inhibition function in TNBC cells. AKT overexpression also promotes MDA-MB-468 cell survival (Fig. 4B-C) and EdU incorporation (Fig. 4D-E) compared to the vector controls.

### FZU-0025-065 represses TNBC cell growth in immunocompromised mice

**Figure 5:**
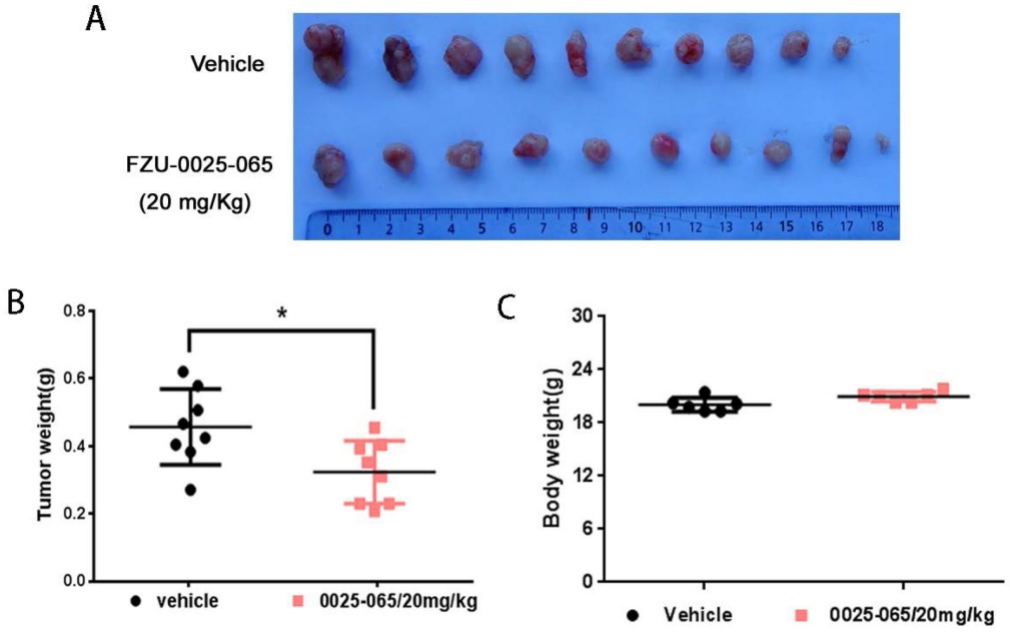
** FZU-0025-065 inhibits TNBC xenograft growth *in vivo*.** (A) FZU-0025-065 suppressed HCC1806 tumor growth in Balb/c nude mice. HCC1806 cells were injected into the fat pat of female Balb/c nude mice. When the average tumor size reached around 50 mm^3^ after inoculation, the mice were randomly distributed into two groups: vehicle control and 20 mg/kg FZU-0025-065. (B) FZU-0025-065 significantly decreased tumor weights compared to the vehicle control (*p<0.05, t-test). (C) Both FZU-0025-065 and vehicle control did not decrease the body weight of the mice. The mice were weighed every other day during the treatment.

Despite the pronounced cytotoxicity effects of FZU-0025-065 exhibited in TNBC cells, we further evaluated the anti-cancer effects of FZU-0025-065 using an *in vivo* xenograft assay. HCC1806 TNBC cells were inoculated to the fat pad of female nude mice. FZU-0025-065 (20 mg/kg) or vehicle was administrated intraperitoneally every other day. As the data shown in Figure 5 and Supplementary Figure 3, FZU-0025-065 significantly suppressed tumor growth compared to vehicle control without affecting the body weight of the mice significantly.

## Discussion

In this study, we examined the cytotoxicity effects of 23 isochromanoindolenine derivatives in three different breast cancer subtype cell lines and selected the most potent anti-cancer compound FZU-0025-065 for further study. FZU-0025-065 has the strongest cytotoxic effects in TNBC cells (Fig. 1). FZU-0025-065 significantly inhibits TNBC cell proliferation and cell cycle progression partially by inhibiting AKT. Finally, we demonstrated that FZU-0025-065 significantly suppressed HCC1806 xenograft tumor growth *in vivo*.

AKT activation is crucial to promote cell cycle and cell proliferation, including phosphorylating and subsequent proteasomal degradation of p21^Cip1/WAF1^ and/or p27^Kip1^ 11-14. We found FZU-0025-065 inhibits TNBC cell proliferation and G0/G1 cell cycle progression. Consistently, FZU-0025-065 suppresses AKT activation and cyclin D1 expression, but promotes p21 and p27 expression (Fig. 3A). Inhibiting AKT activities using a commercial PI3K/AKT inhibitor Alpelisib also suppressed the expression of Cyclins, including Cyclin B1 and Cyclin D1, and decreased TNBC cell viability in a time- and dosage-dependent manner (Supplementary Figure 4). Interestingly, overexpression of constitutive activated AKT partially rescued cell proliferation suppression and cyclin D1 decrease caused by FZU-0025-065 treatment (Fig. 4) without suppressing p21 or p27 expression. Nevertheless, overexpression of constitutive active AKT restored FZU-0025-065 induced cell growth inhibition and DNA synthesis decrease (Fig. 4). These results implicate that FZU-0025-065 at least partially reduce TNBC cell growth through suppressing AKT activation. However, the direct target of FZU-0025-065 and mechanism by which FZU-0025-065 inhibits AKT phosphorylation need further investigation.

It is reported that AKT promotes cyclin B1 expression via facilitating androgen receptor's transcription functions 24. In our study, we found FZU-0025-065 suppresses cyclin B1 expression, and AKT overexpression can partially rescue FZU-0025-065 caused cyclin B1 downregulation (Fig. 3 & 4). Considering cyclin B1 mainly promotes G2/M transition 4, while FZU-0025-065 predominantly induces TNBC G0/G1 cell cycle arrest, we think FZU-0025-065 mainly inhibits TNBC cell growth via suppressing AKT/cyclin D1 axis.

TNBC patients typically receive chemotherapy with anthracycline and cyclophosphamide followed by taxane as standard treatment, approximately one-third of patients achieve pathologic complete response (pCR) and have excellent survival; what's more, TNBC patients had significantly higher pCR rates than non-TNBC patients 25,26, indicating TNBC patients show higher sensitivity to chemotherapy. Besides FZU-0025-065, we also found chemicals, such as Mithramycin A 27, achieve better cytotoxic effects in TNBCs than non-TNBC cells. Although the exact mechanisms have not been fully demonstrated yet, it is reported that the ER- breast cancer subtypes are characterized by the high expression of the proliferation cluster of genes 28; meanwhile, a prognostic index that is predominantly influenced by proliferation genes was shown to predict pCR to doxorubicin/docetaxel primary chemotherapy 29, indicating high proliferation rate of TNBC might facilitate cell sensitivity to chemotherapies, which might be one potential reason. Nevertheless, the exact underline mechanisms are still remained unclear, further studies, such as finding direct targets or genome-wide screening of affected signaling of these compounds in TNBC might help elucidate why TNBC are more sensitive to certain chemotherapeutic drugs.

In summary, we demonstrated that FZU-0025-065 inhibits TNBC cell growth *in vitro* and *in vivo*. The mechanism by which FZU-0025-065 suppresses TNBC cell growth is partially mediated by AKT. Therefore, FZU-0025-065 has the potential to be a novel anticancer agent for human TNBC.

## Materials and Methods

### Reagents, antibodies and plasmids

All compounds, including FZU-0025-065, were designed and synthesized through the last-stage functionalization of tetrahydro-β-carbolines (THβC) via biomimetic oxidative coupling catalyzation, and dissolved in DMSO. PI (propidium iodide, Cat #060M3521V) and sulforhodamine B (SRB) sodium salt (Cat#S9012) were purchased from sigma-Aldrich (St. Louis, MO).

The anti-AKT (#4685S), pAKT1 (#9018S), G3K3β (#9315), STAT3 (#9139S), pSTAT3 (#9131S), p21 (#2947S), α/β-Tubulin (#2148s), and pG3K3β (#9323S) were purchased from Cell Signaling Technology (Danvers, MA). The anti-CDK4 (#sc-749), CyclinB1 (#sc-245), ant GAPDH (#sc-32233) were purchased from Santa Cruz Biotechnology (Dallas, TX). And the anti-CyclinD1 (#A10757) and p27 (#610241) were purchased from ABClonal (Wuhan, CN) and BD Bioscience (San Jose, CA), respectively.

### Cell culture

All cell lines used in this study were purchased from the American Type Culture Collection (ATCC) and validated by STR analysis (Kunming Cell Bank, Kunming Institute of Zoology, Chinese Academy of Sciences). The immortalized breast epithelial cell line 184B5 was maintained in DMEM/Ham's F-12 50/50 medium supplemented with 5% horse serum, 0.5 µg/ml hydrocortisone, 10 µg/ml insulin, 20 ng/ml epidermal growth factor and 0.1 µg/ml cholera enterotoxin. Breast cancer cell lines T47D, HCC1806 and HCC1937 cells were cultured in RPMI-1640 medium with 5% fetal bovine serum (FBS). MDA-MB-231, MDA-MB-468 and SK-BR-3 were cultured in Dulbecco's Modified Eagle's Medium (DMEM) with 5% FBS. MCF7 was maintained in Minimum Essential Medium (MEM) supplied with 5% FBS and 10 µg/ml insulin. SUM149pt was cultured in Ham's F12 supplemented with 5% FBS and 10 µg/ml insulin. All cells were maintained at 37 °C with 5 % CO_2_ in a humidified atmosphere.

### AKT overexpression

The lentiviral pCDH-AKT plasmid was kindly provided by Dr. Binghui Li (Capital Medical University, Beijing, China), and lentiviruses were prepared according to published protocols 30. MDA-MB-468 cells were infected with lentiviruses and the infected cells were selected by 1 µg/ml puromycin and were used for further analysis. Selected cells were maintained in DMEM culture medium supplemented with 5% FBS and 1 µg/ml puromycin, and all experiments were completed using selected populations in 3 passages.

### Cell viability assays

Breast cells were plated with a concentration of 1-4×10^4^ cells/well in 48-well plates. The day after cell seeding, cells were treated with compounds at indicated dosages for 48 hours, followed by being fixed with 10% trichloroacetic acid (TCA) for 30 minutes at room temperature. After being washed with deionized water, fixed cells were then stained with 0.4% Sulforhodamine B (SRB) in 1% acetic acid. Stained cells were washed with 1 % acetic acid and dried. Finally, 10 mM Tris base solution was added, and optical densities were measured at 530 nm in a spectrophotometric plate reader (Biotek, Winooski, VT). Each treatment was set up in triplicate and repeated for three times independently.

### Colony formation assays

Cancer cells were plated in 6-well plate at a density of approximately 5, 000 cells per well. The cells were treated with FZU-0025-065 or controls at indicated dosages on the day after cell seeding. Then cells were cultured for two weeks, and were fixed with 4% paraformaldehyde. Colonies were stained with crystal violet, and finally, 33% acetic acid was added to dissolve the dye, optical results were read at 450 nm using a spectrophotometric plate reader (Biotek, Winooski, VT).

### EdU incorporation assays

Cells were plated in 4-well cell culture slides at a density of 7×10^3^ cells /well. FZU-0025-065 or controls treated cells were then incubated with 20 μM EdU for 4 hours before being fixed by 4% paraformaldehyde for 30 min at room temperature. The EdU-positive cells were detected using cell-Light^TM^ EdU Apollo488 *In vitro* Kit (Cat#S0812, RIBOBIO, Guangzhou, China) following the manufacturer's instructions. Stained cells were visualized and recorded under a fluorescence microscope. The fluorescence positive cells were calculated using the ipwin32 Software.

### Cell cycle analysis

Cancer cells were plated in 6-well plates and treated with FZU-0025-065, FZU-0038-063, or DMSO control at indicated concentrations for 24 h before being collected for cell cycle analysis. In brief, the cells were trypsinized and fixed with 70% ethanol at 4 °C overnight. The fixed cells were stained with propidium iodide (PI) buffer (0.3% NP-40, 0.05 mg/ml PI, 0.5 mg/ml RNase A) in darkness for 30 min at room temperature. The cells were then analyzed on an Accuri C6 flow cytometer (BD bioscience, San Diego, USA) for cell cycle distribution analysis.

### Immunoblotting analysis

Protein extraction and immunoblotting were performed as described before 31. Briefly, cells were collected and lysed using RIPA cell lysis buffer supplied with protease inhibitors. Cell lysates (around 40 μg proteins each sample) were subjected to SDS-PAGE and blotted onto polyvinylidene fluoride (PVDF) membranes. After blocking in 5% evaporated skimmed milk, membranes were incubated with specific primary antibodies at 4℃ overnight, and followed by being incubated with horseradish peroxidase (HRP) conjugated secondary antibodies (Jackson ImmunoResearch Laboratory, West Grove, PA). The signals were visualized by Super ECL Plus reagent and images were taken using an ImageQuant LAS4000 Biomolecular imager (GE, USA).

### Xenograft assays

Animal care and experimental procedures were approved by the institutional ethics committee of Kunming institute of Zoology, CAS. 6-week old female nude athymic mice were purchased from SJA Lab animal Co. Ltd (Hunan, China) and were maintained in specific pathogen-free barrier facilities. HCC1806 cancer cells were collected and suspended in 1×PBS supplied with 20% Matrigel. 1×10^6^ cells/point were inoculated into the fat pat of mice. Tumor size was measured using Vernier calipers once tumors became palpable. Tumor volume was calculated as π/6 (length × width^ 2^). When the average volumes of tumors reached around 50 mm^3^, mice were numbered and divided into 2 groups randomly, which either received intraperitoneal injection of FZU-0025-065 (20 mg/kg) or the vehicle control (10% Solutol® HS 15 dissolved in saline) every two days. Two weeks later, when the biggest tumor reached 1.5 cm in diameter, all mice were sacrificed in accordance with KIZ Animal Rights Committee guidelines, and tumors were surgically dissected for analysis.

### Statistical analysis

All experiments were repeated at least three times. The data were pooled and expressed as the mean ± standard deviation and analyzed by Student's t-test. P values less than 0.05 were considered as significant.

## Supplementary Material

Supplementary figures and tables.
