# Supplementary material for: Isochromanoindolenines suppress triple-negative breast cancer cell proliferation partially via inhibiting Akt activation
Source: Int J Biol Sci. 2021 Mar 2;17(4):986–94. doi: 10.7150/ijbs.48170 (PMC8040301; doi:10.7150/ijbs.48170)
Supplement: Supplementary file 1 — Supplementary figures and tables. [file ijbsv17p0986s1.pdf]

Supplementary figure legends

Supplementary figure 1. FZU-0025-065 moderately induce apoptosis in TNBC cells.

(A) FZU-0025-065 induces apoptosis in HCC1806, but not in MDA-MB-468 cells. FZU-0025-065, FZU-0038-063 or DMSO treated cells were collected for Annexin V staining. The quantitative results of HCC1806 and MDA-MB-468 were shown on the right.

(B) HCC1806 and MDA-MB-468 cells were treated with 9 μM FZU-0025-065, FZU-0038-063 or DMSO for indicated time. The cells were then collected for WB analysis. β-actin was detected as the loading control.

Supplementary figure 2. FZU-0025-065 suppresses TNBC cell cycle progression partially through inhibiting AKT singaling.

Ectopic overexpression of AKT partially rescued FZU-0025-056 caused reduction of cyclin D1 and CDK4, it also partially rescued FZU-0025-056 induced up-regulation of p21 and p27.

Supplementary figure 3. FZU-0025-065 suppresses HCC1806 cell growth in xenograft mouse model

HCC1806 cells were injected into the fat pat of female Balb/c nude mice. When the average tumor size reached about 50 mm^3 after inoculation, the mice were randomly distributed into two groups: vehicle control and 20 mg/kg FZU-0025-065. Tumors were measured with a caliper every other day and were collected 2 weeks after drug treatment.

Supplementary figure 4. Alpelisib suppresses AKT activation and regulates cell cycle associated proteins' expression

(A) Alpelisib suppresses AKT activation.

(B) Alpelisib inhibits TNBC cell survival. HCC1806 and MDA-MB-468 cells were

treated with Alpelisib, FZU-0025-065 or DMSO control for indicated time and at indicated dosages. Cells were then fixed for SRB assay.

(C-D) Alpelisib suppresses the expression cyclin B1, cyclin D1 and CDK4. HCC1806 and MDA-MB-468 cells were treated with indicated compounds or DMSO control for indicated time and at indicated dosages. α,β-tubulin were detected as loading control.

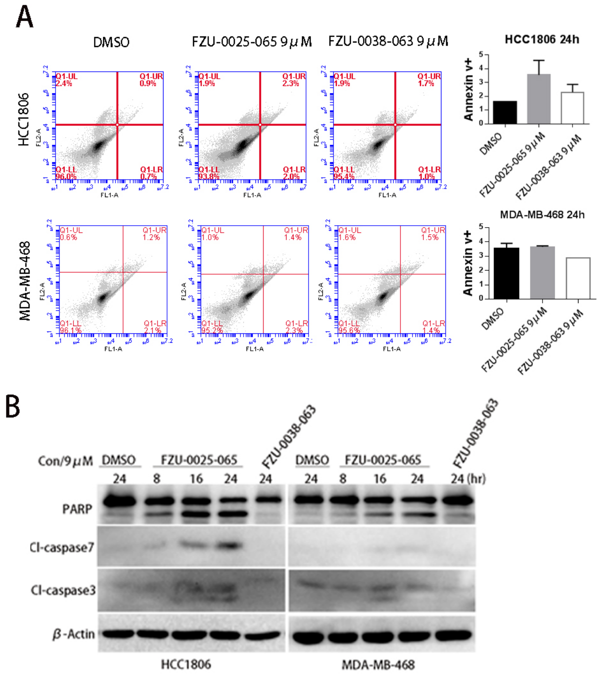

A
DMSO
FZU-0025-065 9μM
FZU-0038-063 9μM
HCC1806
MDA-MB-468
Q1-UL 2.4%
Q1-UR 0.9%
Q1-LL 96.0%
Q1-LR 0.7%
Q1-UL 1.9%
Q1-UR 2.3%
Q1-LL 93.8%
Q1-LR 2.0%
Q1-UL 1.9%
Q1-UR 1.7%
Q1-LL 95.4%
Q1-LR 1.0%
Q1-UL 0.6%
Q1-UR 1.2%
Q1-LL 96.1%
Q1-LR 2.1%
Q1-UL 1.0%
Q1-UR 1.4%
Q1-LL 95.2%
Q1-LR 2.3%
Q1-UL 1.6%
Q1-UR 1.5%
Q1-LL 95.6%
Q1-LR 1.4%
FL1-A
FL2-A
HCC1806 24h
MDA-MB-468 24h
Annexin v+
DMSO
FZU-0025-065 9μM
FZU-0038-063 9μM
B
Con/9μM
DMSO
FZU-0025-065
FZU-0038-063
24
8
16
24 (hr)
PARP
Cl-caspase7
Cl-caspase3
β-Actin
HCC1806
MDA-MB-468

Figure S1

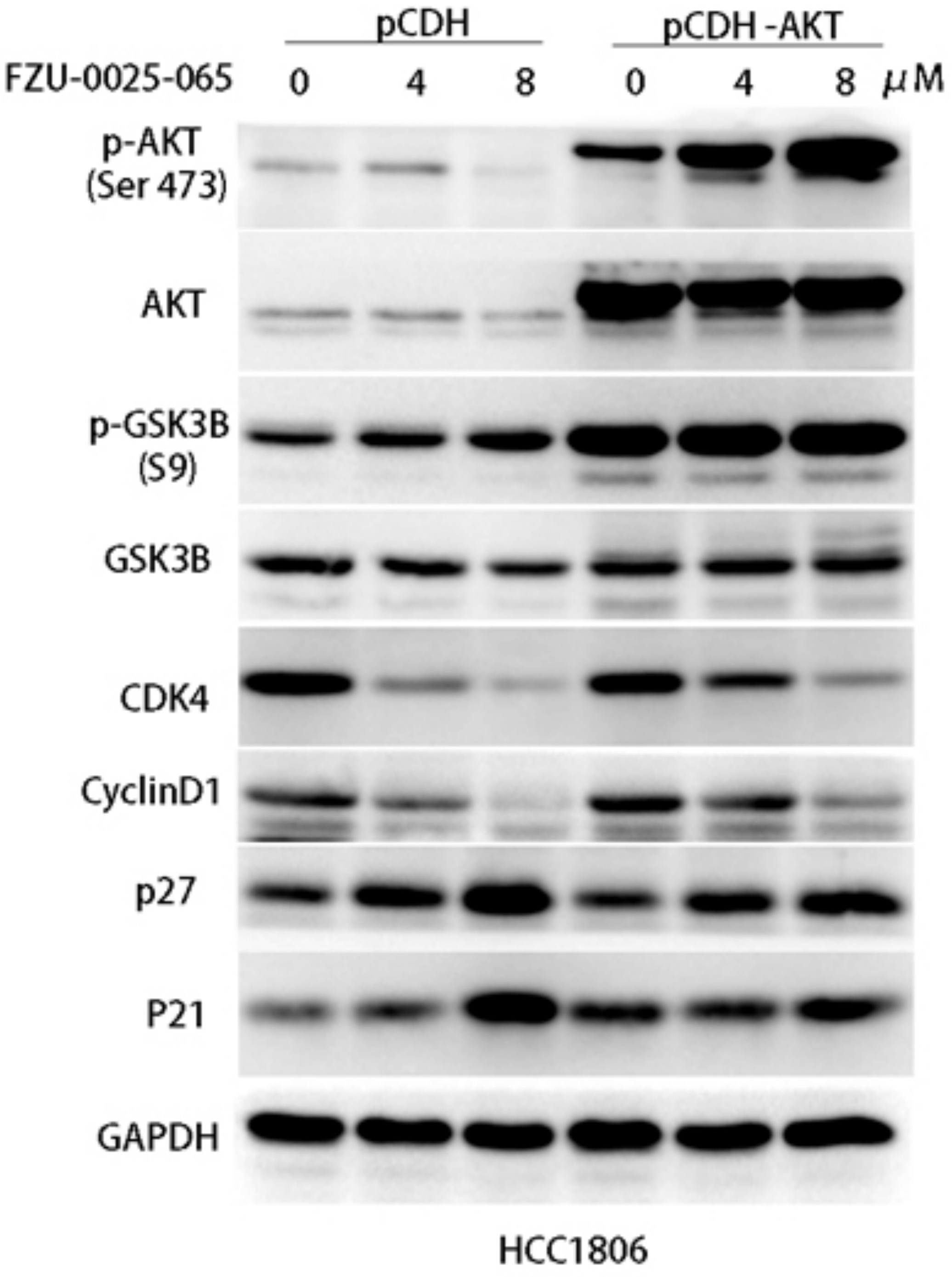
pCDH
pCDH -AKT
FZU-0025-065
0 4 8 0 4 8 μM
p-AKT (Ser 473)
AKT
p-GSK3B (S9)
GSK3B
CDK4
CyclinD1
p27
P21
GAPDH
HCC1806

Figure S2

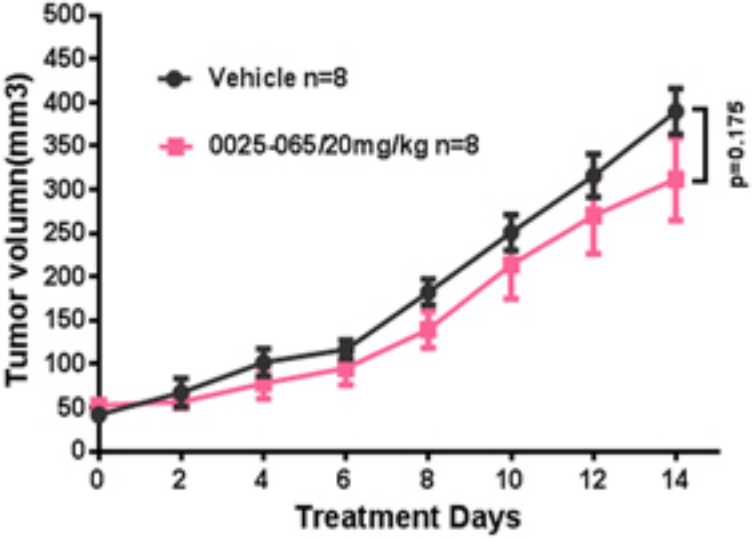
500
450
400
350
300
250
200
150
100
50
0
Tumor volumn(mm3)
Vehicle n=8
0025-065/20mg/kg n=8
p=0.175
0
2
4
6
8
10
12
14
Treatment Days

Figure S3

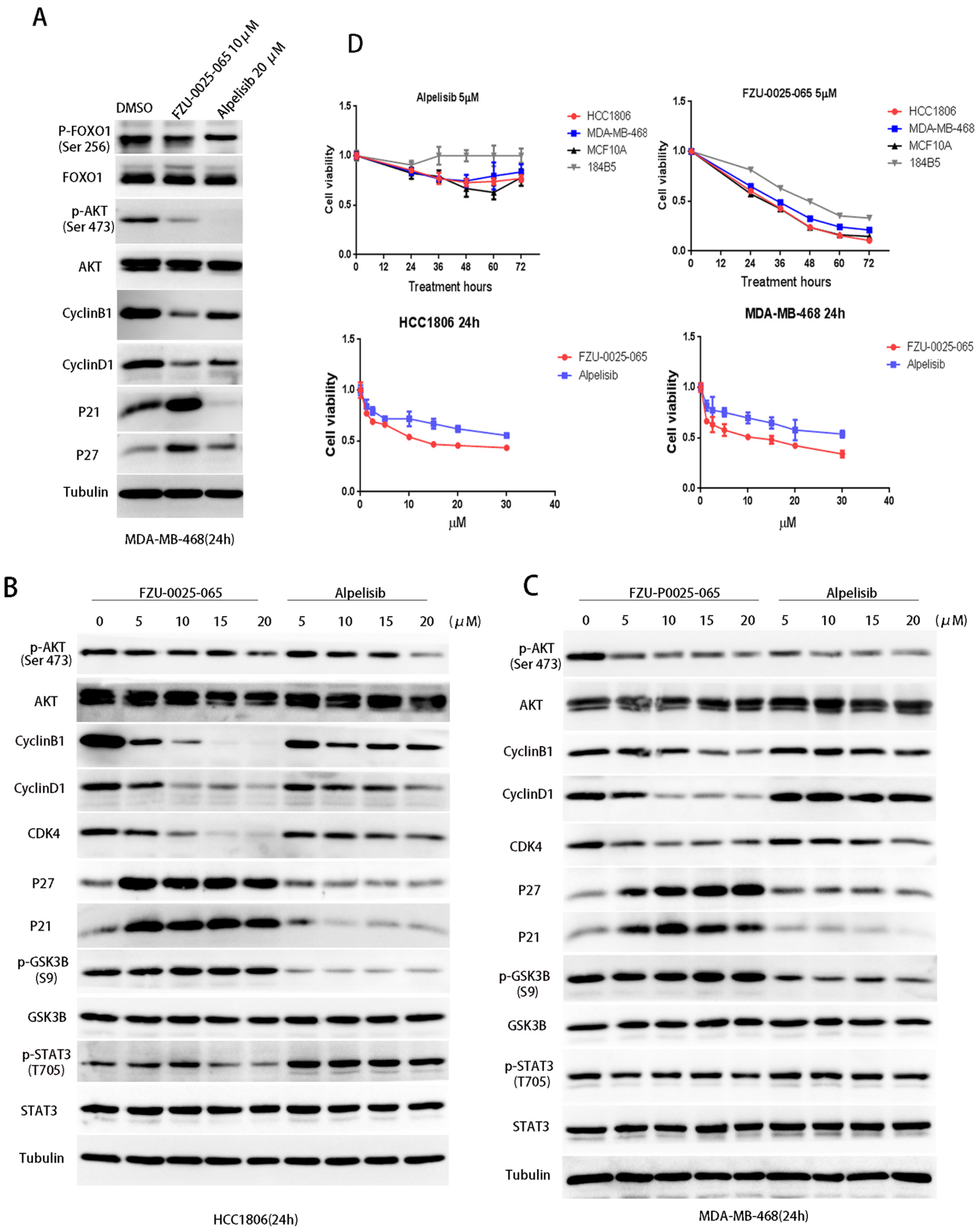
A
DMSO
FZU-0025-065 10 μM
Alpelisib 20 μM
P-FOXO1 (Ser 256)
FOXO1
p-AKT (Ser 473)
AKT
CyclinB1
CyclinD1
P21
P27
Tubulin
MDA-MB-468(24h)
D
Alpelisib 5μM
Cell viability
Treatment hours
HCC1806
MDA-MB-468
MCF10A
184B5
FZU-0025-065 5μM
HCC1806 24h
FZU-0025-065
Alpelisib
μM
MDA-MB-468 24h
B
FZU-0025-065
Alpelisib
0 5 10 15 20 5 10 15 20 (μM)
p-AKT (Ser 473)
AKT
CyclinB1
CyclinD1
CDK4
P27
P21
p-GSK3B (S9)
GSK3B
p-STAT3 (T705)
STAT3
Tubulin
HCC1806(24h)
C
FZU-P0025-065
Alpelisib
0 5 10 15 20 5 10 15 20 (μM)
MDA-MB-468(24h)

Figure S4

Supplementary Table 1. Structure of isochromanoindolenine compounds

Compound ID	Formula	Molecular Weight	Chemical Structure	Solubility
FZU-0025-065	C 25 H 21 ClN 2 O 6 S	512.96	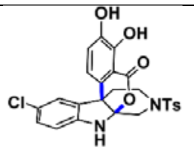	DMSO
FZU-0038-063	C18H17ClN2O2S	360.07	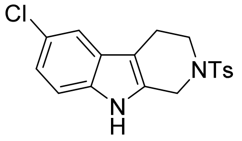	DMSO
FZU-0025-044	C 24 H 20 N 2 O 6 S	464.49	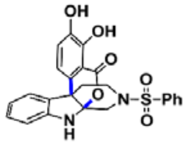	DMSO
FZU-0025-046	C 27 H 26 N 2 O 6 S	506.57	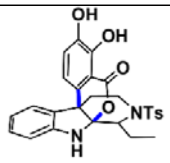	DMSO
FZU-0025-048	C 19 H 18 N 2 O 6 S	402.42	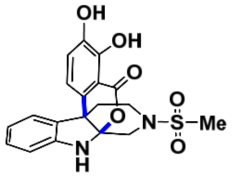	DMSO
FZU-0025-101	C 19 H 17 ClN 2 O 6 S	436.86	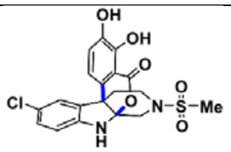	DMSO
FZU-0038-132	C 20 H 18 N 2 O5	366.37	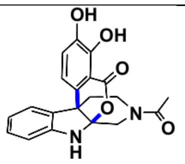	DMSO
FZU-0025-102	C 20 H 17 ClN 2 O5	400.82	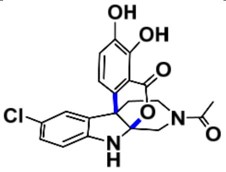	DMSO
FZU-0038-130	C 20 H 18 N 2 O6	382.37	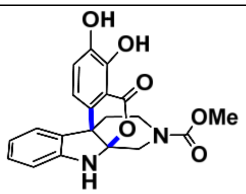	DMSO

FZU-0025-103	C 20 H 17 ClN 2 O6	416.81	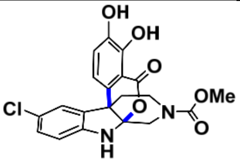	DMSO
FZU-0021-263	C 25 H 22 N 2 O 6 S	478.52	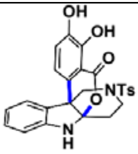	DMSO
FZU-0025-066	C 25 H 21 FN 2 O 6 S	496.51	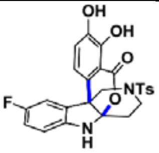	DMSO
FZU-0025-070	C 26 H 24 N 2 O 6 S	492.55	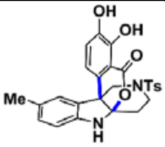	DMSO
FZU-0025-097	C 25 H 21 FN 2 O 6 S	496.51	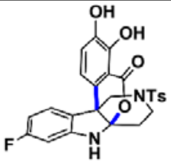	DMSO
FZU-0025-096	C 26 H 24 N 2 O 6 S	492.55	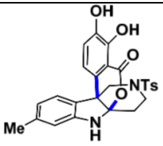	DMSO
FZU-0025-079	C 24 H 20 N 2 O 6 S	464.49	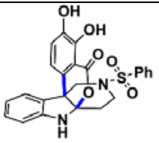	DMSO
FZU-0025-080	C 24 H 19 ClN 2 O 6 S	498.93	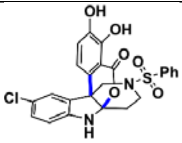	DMSO
FZU-0025-081	C 25 H 22 N 2 O 7 S	494.52	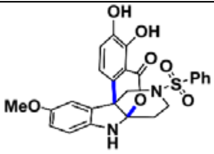	DMSO
FZU-0025-082	C 20 H 18 N 2 O5	366.37	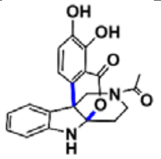	DMSO
FZU-0025-083	C 20 H 17 ClN 2 O5	400.82	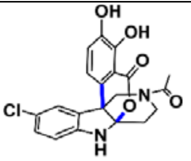	DMSO

FZU-0025-085	C 20 H 18 N 2 O6	382.37	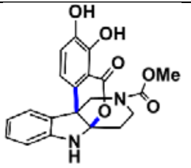	DMSO
FZU-0025-086	C 20 H 17 ClN 2 O1	416.81	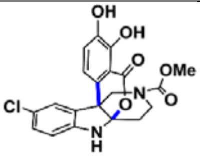	DMSO
FZU-0021-258	C 23 H 24 N 2 O6	424.45	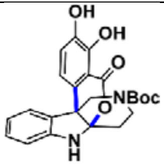	DMSO
FZU-0025-088	C 26 H 21 N 3 O5	455.47	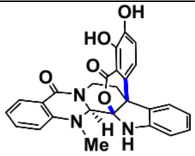	DMSO
